# Potential and Challenges of Improving Photosynthesis in Algae

**DOI:** 10.3390/plants9010067

**Published:** 2020-01-03

**Authors:** Valeria Vecchi, Simone Barera, Roberto Bassi, Luca Dall’Osto

**Affiliations:** Dipartimento di Biotecnologie, Università di Verona, Strada Le Grazie 15, 37134 Verona, Italy; valeria.vecchi@univr.it (V.V.); Simone.barera@univr.it (S.B.); roberto.bassi55@gmail.com (R.B.)

**Keywords:** photosynthesis, microalgae, biomass productivity, NPQ, light-harvesting, complex PSII, RuBisCO, renewable energies, strain domestication

## Abstract

Sunlight energy largely exceeds the energy required by anthropic activities, and therefore its exploitation represents a major target in the field of renewable energies. The interest in the mass cultivation of green microalgae has grown in the last decades, as algal biomass could be employed to cover a significant portion of global energy demand. Advantages of microalgal vs. plant biomass production include higher light-use efficiency, efficient carbon capture and the valorization of marginal lands and wastewaters. Realization of this potential requires a decrease of the current production costs, which can be obtained by increasing the productivity of the most common industrial strains, by the identification of factors limiting biomass yield, and by removing bottlenecks, namely through domestication strategies aimed to fill the gap between the theoretical and real productivity of algal cultures. In particular, the light-to-biomass conversion efficiency represents one of the major constraints for achieving a significant improvement of algal cell lines. This review outlines the molecular events of photosynthesis, which regulate the conversion of light into biomass, and discusses how these can be targeted to enhance productivity through mutagenesis, strain selection or genetic engineering. This review highlights the most recent results in the manipulation of the fundamental mechanisms of algal photosynthesis, which revealed that a significant yield enhancement is feasible. Moreover, metabolic engineering of microalgae, focused upon the development of renewable fuel biorefineries, has also drawn attention and resulted in efforts for enhancing productivity of oil or isoprenoids.

## 1. Introduction

### 1.1. Why Study Photosynthesis in Microalgae?

Oxygenic photosynthesis is the process by which photoautotrophs capture sunlight efficiently and converts it into organic molecules and biomass with an efficiency which is, instead, variable, depending on species and environmental conditions [1]. Oxygenic photosynthetic organisms, namely plants, algae and cyanobacteria, store into biomass the solar energy that reaches the Earth’s surface at a rate of 120,000 terawatt/year (TW-y) [2]. The current global energy demand of 14.9 TW-y, although it is projected to increase to 23.4 TW-y by 2030, yet falls greater than three orders of magnitude behind the solar energy on Earth. Therefore, exploitation of this potential by culturing photoautotrophs could satisfy at least part of the energy required for anthropic activities. 

Among photosynthetic organisms, cyanobacteria and eukaryotic microalgae are the most promising feedstocks for the sustainable production of bulk bio-based materials such as food, feed, fuel and high-value metabolites; moreover, they can be used for wastewater treatments and in mitigation processes for CO_2_-emissions [3]. Algae can grow autotrophically, heterotrophically or mixotrophically in massive cultures for industrial purposes, in either open ponds or closed photobioreactors (PBRs). In contrast with land plants, algae do not require arable land and need far less fresh water for their growth. Moreover, the culture biomass devoid of stems and roots, which consumes metabolic energy, is fully photosynthetically active. Finally, biomass productivity is far less affected by the seasonal cycle [3]. However, while microalgae represent a promising source of valuable bio-based products, an optimization of cultivation technologies is required in order to enhance growth rates and cell densities at saturation, thus making the process profitable [4]. Indeed, productivity in photobioreactors is reduced by the inefficient light-to-biomass conversion, that leads to a photosynthetic efficiency significantly lower than the theoretical maximum of 9–10%, corresponding to ~80 g of biomass/m^2^/day or 280 ton/ha/year [5]. In the industrial scale PBRs, algae light conversion yield falls between 3% and 5% [5]. Filling the gap that originates from light-use inefficiency, and that makes the controlled cultivation of microalgae still far from being commercially viable, is therefore essential. Comprehension of the mechanism regulating photosynthesis will allow researchers to identify the targets for genetic improvement and ultimately to enhance biomass yield, thus counterbalancing the costs for cultivation systems and downstream biomass processing.

### 1.2. Microalgal Species of Interest for Research on the Regulatory Mechanisms of Photosynthesis

Eukaryotic microalgae are classified according to their pigment content into *Rhodophyta* (red algae), *Chrysophyceae* (golden algae), *Phaeophyceae* (brown algae) and *Chlorophyta* (green algae) [6]. *Chlorophyta* includes most genera currently employed for biotechnological applications [3]. The best studied green microalgal species is certainly the model organism *Chlamydomonas reinhardtii*. The major reasons for this preeminent position in photosynthesis research resides in its haploid genetic organization, allowing the mutant phenotypes to be detected at the first generation without the need for segregation; moreover, sexual reproduction can be induced by modulating the growth conditions, it can be transformed in all its genomes (nuclear, chloroplastic and mitochondrial), and it is mixotrophic, thus allowing for the isolation of mutants with impaired photosynthesis [7]. Finally, a short life cycle makes it a good platform to study light-to-biomass conversion efficiency and to optimize photosynthesis [8]. Besides *Chlamydomonas*, genetic tools have been developed for other species of green algae, which have an exploitation potential for high-value chemicals production [3]. Among those, *Chlorella zofigiensis* accumulates high-value carotenoids and has high biomass and lipid productivity [9]; several species belonging to the genus *Chlorella* are of interest for human health supplements [10] and biofuel production [11]. Moreover, domestication strategies have been developed in *C. sorokiniana* to generate mutant strains with enhanced biomass productivity [12]. In other *Chlorophyta*, limitations related to the lack of optimized genetic tools still exist, and particularly concern strains relevant for industrial applications: *Dunaliella salina*, extensively cultured in open ponds and photobioreactor for β-carotene [13] and lipids production [14] and *Haematococcus pluvialis*, an industrial source of astaxanthin [15]. Members of the *Nannochloropsis* genus, and the diatom *Phaeodactylum tricornutum*, all belonging to Heterokonta, are obligate photoautotrophs that have been intensively characterized, and are also well-developed models for studying microalgal molecular physiology and genetic engineering. The photosynthetic mechanisms of different species such as *Nannochloropsis gaditana*, *N. oceanica* or *N. oculata* have been investigated because of their unique photosynthetic architecture among Heterokonta, characterized by Chl *a* as the only primary pigment and high content of violaxanthin and vaucheriaxanthin [16]; moreover, light regimes and nutrient starvation induce rapid *triacylglycerols* (TAGs) biosynthesis in these oleaginous strains, that are therefore considered promising for biodiesel production [17]. *Phaeodactylum tricornutum*, a species with a fully sequenced genome, is interesting for its high lipid content and for a peculiar light-harvesting system, binding the xanthophyll fucoxanthin (Fx), Chls *a* and *c* [18,19].

In this review we mainly focused on green microalgae and diatoms, citing other species whenever it is considered relevant.

## 2. Photosynthesis

**Figure 1:**
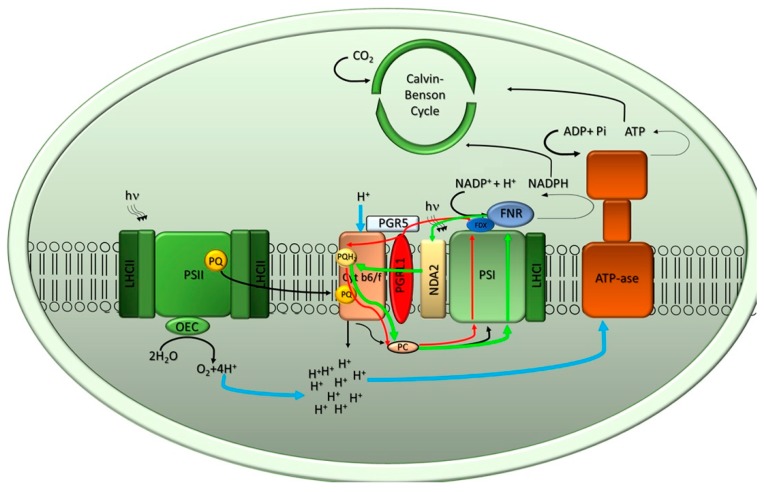
Schematic representation of photosynthetic electron transport. Arrangement of Photosystem I (PSI), Photosystem II (PSII), cytochrome b6f and adenosine triphosphate (ATP) synthase complexes within the thylakoid membranes is shown. The light-driven water splitting reaction leads to O_2_ evolution and originates linear electron transport (LET), indicated with black arrows, from water to nicotinamide adenine dinucleotide phosphate (NADP^+^), which is coupled to proton translocation from stroma into the luminal side of thylakoids during the light phase. The electrochemical gradient formed is used by the ATP synthase to produce ATP from Adenosine diphosphate (ADP) and Pi in the stroma. The NADPH and ATP formed during the light phase drive the Calvin–Benson–Bassham cycle reactions in the stroma. Two pathways of cyclic electron transport (CET) around PSI are indicated with red (Ferredoxin-dependent pathway) and green (NDA2-dependent pathway) arrows, respectively.

In both green algae and higher plants, the process of oxygenic photosynthesis can be divided into light and dark phases. In the former, photons are absorbed and utilized to drive Linear and Cyclic Electron Transfer (LET and CET, respectively), to form adenosine triphosphate (ATP) and the reduced form of Nicotinamide adenine dinucleotide phosphate (NADPH), which power the Calvin–Benson–Bassham cycle to produce carbohydrates in the dark phase (Figure 1).

### 2.1. The Light Phase of Photosynthesis

The linear electron transport (LET) reaction starts with the water-splitting complex Photosystem II (PSII), that captures sunlight and utilizes excitation energy to oxidize water molecules into protons (H^+^) and molecular oxygen (O_2_). The electrons removed from water are transferred via the Plastoquinone (PQ) pool to the Cytochrome *b*_6_*f* (Cyt *b*_6_*f*) complex and then utilized to translocate protons across the thylakoid membrane. The cytochrome *f* subunit reduces the soluble electron carriers’ plastocyanin (PC), the electron donors of PSI. Absorption of photons by Photosystem I (PSI) promotes oxidation of its reaction centre (RC) P700. The electron removed by the oxidation event finally reduces ferredoxin (FDX) and the electron hole in P700^+^ is filled by electrons from PC [20], while at the stromal side the ferredoxin NADP^+^ reductase (FNR) transfers the electrons from FDX to NADP^+^ to yield NADPH + H^+^. This electron transport is coupled to the build-up of a proton gradient across the thylakoid membrane, with contributions from water splitting and PQH_2_ oxidation by the Cyt *b*_6_*f*. The return of protons to the stromal compartment is coupled to ATP synthesis [20]. 

The ATP:NADPH ratio is regulated by modulating LET and CET, the latter being the reaction which reduces PQ with excess reducing equivalent from FDX or NADPH [21] (Figure 1). In *C. reinhardtii*, two CET pathways around PSI are suggested: The NADPH-dependent pathway involves type II NAD(P)H-dehydrogenase (NDA2), which recycles electrons from PSI into the intersystem chain via NADPH [22]. The secondary FDX dependent pathway is mediated by two proteins: PROTON GRADIENT REGULATION 5 (PGR5) and PGR5-LIKE PHOTOSYNTHETIC PHENOTYPE 1 (PGRL1) [23] (Figure 1).

#### 2.1.1. Light-Harvesting Systems: PSI-LHCI and PSII-LHCII Supercomplexes Organization in Microalgae

Capture of light energy by both photosystems, which drives charge separation in RC and fuels LET and CET, is enhanced by pigment-binding proteins, the light-harvesting complexes (LHC). Various LHCs form the peripheral antenna system in both photosystems [24]. While RC subunits were strongly conserved, the antenna complexes diversified through evolution [24,25], yet maintained a common architecture [26,27]. The most represented member is the major antenna LHCII, a 22 kDa polypeptide which binds 14 chlorophylls (Chl) *a* and *b*, and four xanthophylls (Lutein, Neoxanthin, Violaxanthin and, upon high light exposure, Zeaxanthin) [28] (LHC)-like antenna proteins, which were present in a cyanobacterial ancestor, carried out photoprotective functions [29], while they later evolved into isoforms fulfilling either light-harvesting or energy-dissipative responses. The LHC superfamily consists of some 30 proteins, the most conserved being the subunits of PSI and PSII through the *Chlorophyta* [30], which have pre-eminently a light-harvesting role, while the light-harvesting complex stress-related (LHCSR) subunits have an energy-dissipative role, enabling photoprotection in excess light (EL) conditions through the non-photochemical quenching (NPQ) mechanism [31] (see Section 2.3). In *C. reinhardtii*, the LHCI subunits, forming the PSI peripheral antenna system, and the monomeric subunits of the PSII supercomplex Lhcb4 (CP29) and Lhcb5 (CP26), are the most conserved antenna proteins. Trimeric LHCIIs, the major antennae of photosynthetic membranes of *C. reinhardtii*, are encoded by *Lhcbm* genes (*Lhcbm1*–*9*).

##### PSII-LHCII

**Figure 2:**
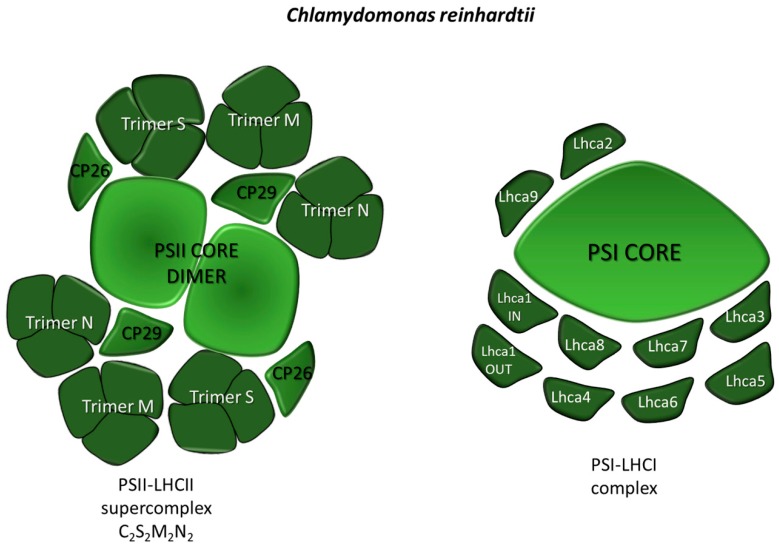
Supramolecular organization of PSII-LHCII and PSI-LHCI supercomplexes in the model alga *Chlamydomonas reinhardtii*. The schematic representations are based upon data from [33] for PSII-LHCII and from [36] for PSI-LHCI. The core complexes of both PSs are shown in light green while the antenna complexes are shown in dark green.

The core complex of PSII is highly conserved in all organisms and consists of 40 different protein subunits. The RC is composed by subunits D1, D2 and cytochrome *b*_559_ and hosts P680, the PSII RC where the primary charge separation event occurs. Light-dependent transfer of reducing equivalents to PQ leads to P680^+^ formation. The positive charges accumulated by four events of charge separation drive the water splitting reaction within the oxygen evolving complex (OEC), composed by the extrinsic polypeptides PsbO, PsbQ, PsbP and PsbR. Chl *a*- and β-carotene-binding inner antennae CP43 and CP47 enlarge the light harvesting capacity of the supercomplex. The PSII core is organized into dimers (C2), which, in turn coordinate a peripheral antenna system (see above). In higher plants, this LHC system is made of two layers: The inner, composed by the monomeric LHC proteins CP24, CP26 and CP29 [32], which are bound, respectively, to the CP43 and CP47 core subunits, and the outer layer is made by the trimeric LHCII complexes [33]. In *C. reinhardtii*, the largest PSII-LHCII supercomplex characterized contains three LHCII trimers (named S, M and N) per monomeric core, and it is characterized by the absence of the monomeric antennae protein CP24. In mosses and higher plants, the N trimer has been substituted for by an additional monomeric LHC, CP24 (Lhcb6), LhcbM1, LhcbM2/7 and LhcbM3, which are the major components of LHCII trimers in the PSII supercomplex of *C. reinhardtii* [34] (Figure 2). Recently, Shen and co-workers reported a cryo-electron microscopy structure of a complete, C_2_S_2_M_2_N_2_-type PSII–LHCII supercomplex from *C. reinhardtii* at 3.37-A resolution. The high-resolution structure allowed not only locating the LHCII trimers in the complex, but also the plausible energy transfer pathways from the peripheral antennae to the PSII core. Moreover, a number of small core subunits (PsbE, PsbF, PsbH, PsbI, PsbJ, PsbK PsbL, PsbM, PsbTc, PsbW, PsbX and PsbZ) has been elucidated [35].

##### PSI-LHCI

The organization of PSI-LHCI of *C. reinhardtii* was investigated by negative stain electron microscopy and single particle analysis, which revealed that the supercomplex is larger but less stable than that from higher plants. The Lhca1-9 antenna proteins loosely bind to the core, where this can explain the large variation in antenna composition of PSI-LHCI from *C. reinhardtii* found in the literature. The isolation of several PSI-LHCI supercomplexes with different antenna size allowed to precisely determine the position of Lhca2 and Lhca9 proteins and led to a model of whole PSI-LHCI supercomplex antenna organization [36]. Moreover, a megacomplex constituted by a Cyt *b*_6_*f* interacting with the PSI-LHCI complex was identified under anaerobic conditions, a treatment that promotes CET in *C. reinhardtii* [37]. More recently, the structure of *C. reinhardtii* PSI–LHCI supercomplex has been solved by cryo-electron microscopy, showing that up to ten LHCIs are associated with the PSI core [38] (Figure 2).

### 2.2. The Dark Phase of Photosynthesis

In green algae and higher plants, the carbon dioxide reduction occurs in the dark phase of photosynthesis, which also occurs in the light, and is powered by the NADPH and ATP from the light phase. The whole process can be described with the general reaction:

$$CO_{2}+4H^{+}+\;4e^{-}\overset{\begin{array}{c} 2NADPH+H+3ATP\\ Enzymes \end{array}\;}{\to }\;\left(CH_{2\;}O\right)+\;H_{2}O$$

The entire process of carbon fixation, discovered by Calvin, Benson and Bassham in the early 1950s, requires two molecules of NADPH and three of ATP for each CO_2_ fixed into sugars. This energy complement is supplied by the absorption of eight photons in the light phase.

#### 2.2.1. Dark Reactions of Photosynthesis: The Calvin-Benson-Bassham Cycle

**Figure 3:**
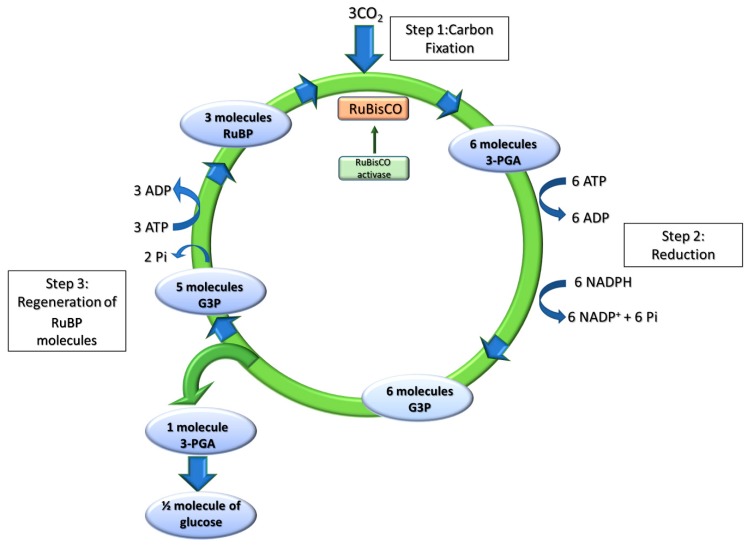
The Calvin–Benson–Bassham cycle (CBBc) reactions. The CBBc has three stages. In stage 1, the enzyme RuBisCO incorporates 3 CO_2_ molecules into the 5-carbon sugar ribulose-1,5-bisphosphate (RuBP) to form 6 molecules of 3-phosphoglycerate (3-PGA). In stage 2, 6 molecules of 3-PGA are converted into 6 molecules of Glyceraldehyde-3-P (G3P) by using 6 molecules of ATP and 6 molecules of NADPH as reducing power. In stage 3, RuBP is regenerated so that the cycle can continue. Stage 3 includes a complex series of reactions combining 3-, 4-, 5-, 6-, and 7-carbon sugar phosphates, which are not explicitly shown in the diagram.

The conversion of CO_2_ into sugar (or other compounds) is performed by three distinct phases (carboxylation, reduction and regeneration phases) within the Calvin–Benson–Bassham cycle (CBBc) (Figure 3). During the carboxylation phase, one molecule of CO_2_ is added to the 5-carbon sugar ribulose bisphosphate (RuBP) by the enzyme ribulose bisphosphate carboxylase/oxygenase (RuBisCO) to form two molecules of phosphoglycerate (3-PGA). The enzyme is controlled by the RuBisCO-activase, which carboxylates a Lys residue in the presence of the substrate CO_2_, thus preventing wasteful reaction with O_2_ under CO_2_-depleted conditions. The subsequent reduction phase catalyses the conversion of 3-PGA into 3-carbon (Triose-P) products glycerhaldeide-3-P (G3P) in two steps, by consuming ATP and NADPH. The regeneration phase restores the initial RuBP reactant from Triose-P, through a complex series of reactions which involves eight distinct enzymes (Figure 3), including transketolase and transaldolase, yielding 5-carbon sugars from 6-carbon plus 3-carbon sugar intermediates, and the sedoheptulose-1,7-bisphosphatase that catalyses the de-phosphorylation of sedoheptulose-1,7-bisphosphate to yield sedoheptulose-7-phosphate. sedoheptulose-1,7-bisphosphatase activity shows a strong correlation with the rate of photosynthetic carbon fixation, thus controlling carbon flux [39]. Under EL conditions, the RuBisCO activity rate becomes limiting, and the rate of synthesis of ATP/NADPH from light reactions exceeds their use by CBBc. Depletion of ADP limits ATPase activity in protons’ return to the stroma compartment, leading to lumen iper-acidification and triggering excess energy dissipation reactions. This is further enhanced by NADPH accumulation, since CET activation further acidifies the lumen.

#### 2.2.2. RuBisCO

In cyanobacteria and plants, along with red, brown and green algae, RuBisCO is found as a large protein complex with a hexadecameric quaternary structures consisting of eight 55-kDa large (L) subunits and eight 15-kDa small (S) subunits (L_8_S_8_). The RuBisCO crystal structure was determined on complexes purified from *Spinacia oleracea*, *Nicotiana tabacum* [40] and the cyanobacterium *Synechococcus* [41]. In green algae the crystal structure of *C. reinahardtii* at 1.4 A resolution [42] showed high similarity to the L_8_S_8_-RuBisCO enzyme assembly from *Spinacia oleracea*. Since RuBisCO evolved in atmosphere with a higher concentration of CO_2_ respect to the current one, it has low affinity for CO_2_ and low substrate specificity. Thus, it accepts oxygen as substrate at low CO_2_, which leads to the loss of fixed carbon as a consequence of feeding a photorespiratory cycle with phosphoglycolate. Although the photorespiratory (C2) cycle recovers part of phosphoglycolate into phosphoglycerate, this reduces the overall light-to-biomass conversion efficiency. In both green algae and higher plants, the ability to fix CO_2_ depends in part upon the properties of RuBisCO: While RuBisCO isolated from a few species of red algae have three times higher substrate specificity vs. that from C3 crop species [43], in most of photoautotrophs RuBisCO is operating at no more than 30% of its capacity under standard atmospheric conditions (21% O_2_, 0.04% CO_2_). Indeed, the chloroplastic abundance of this protein is extremely high. To overcome this drawback, many photosynthetic organisms have developed different systems to increase the level of CO_2_ at the catalytic site in order to enhance the carboxylation while disfavouring the oxygenation reaction. Microalgae absorb HCO_3_^–^ ions, which must be converted to CO_2_ before the carbon fixation takes place. Moreover, in green algae RuBisCO is compartmentalized into carbon-concentration sub-compartments of the chloroplast, called pyrenoids, which have been purified from *C. reinhardtii* [44] and shown they consist primarily of RuBisCO. In other algae types including red algae, carboxysomes are present as large molecular architectures including carbonic anhydrase together with RuBisCO, where CO2 level is increased from carbonic anhydrase activity to limit photorespiration and enhance photosynthetic yield [45].

### 2.3. Dynamics of the Photosynthetic Apparatus in Response to Environmental Conditions: Photoprotective Mechanisms

During evolution, photosynthetic organisms are said to have adapted to a wide range of habitats with an extreme variability of light irradiances, water and nutrient abundance and temperature. Abiotic stresses such as drought or nutrient deprivation easily decrease the maximum photosynthetic yield of algae, thus environmental conditions can exacerbate EL stress. In this condition, the energy absorbed exceeds the rate of its utilization by downstream reactions, increases the concentration of Chl-excited singlet states (^1^Chl *), thus the probability of Chl triplet states (^3^Chl *) formation together with the release of singlet oxygen (^1^O_2_), a reactive oxygen species (ROS). It comes that a mechanism to dissipate the excitation energy absorbed in excess, is required. Experiencing EL conditions activate the Non-Photochemical Quenching (NPQ) process. This can be experimentally observed as a decrease of fluorescence emitted by PSII upon exposure to over-saturating light. NPQ arises from a number of processes in the thylakoid membranes, and several major components of NPQ can be identified based on the kinetics curves of the relaxation of PSII fluorescence [46]. The fastest component, immediately triggered upon exposure to EL, is the energy-dependent quenching (qE), which relaxes within approx. one minute upon switching actinic light off. 

State transitions (STs) represent changes in the relative antenna sizes of photosystems [47], however although this fluorescence decline (called qT) has been included in NPQ, it is caused by PSI RC activity, and therefore is of the photochemical type. An additional quenching component, that rises and relaxes at a longer time scale than qE, is called qZ [48]: This is found in some algae species in which a zeaxanthin-dependent enhancement of NPQ is observed [49]. The slowest component, named qI, develops under long lasting (several hours) high light stress [46].

The qE response is dependent on a low lumenal pH and requires LhcsR, Chl-xanthophyll-binding proteins found in eukaryotic algae and mosses [50] and is replaced by the non-pigmented protein PsbS in higher plants [51,52]. In *C. reinhardtii*, LhcsR proteins are encoded by three highly homologous genes *LhcsR1*, *LhcsR3.1* and *LhcsR3.2*, while PsbS by two closely linked *PsbS1* and *PsbS2* genes. Both PsbS and LhcsR proteins harbour protonatable residues exposed to the luminal side, which detect low pH and activate the heat dissipation of energy absorbed in excess [53,54]. In *C. reinhardtii*, accumulation of gene products involved in qE is induced by signals such as high light, blue light and UV light via increased expression of genes encoding for LhcsR and PsbS. By a forward genetics approach, SPA1 and CUL4 have been identified as components of a putative green algal E3 ubiquitin ligase complex, as critical factors in a signalling pathway that controls light-regulated expression of the dissipative response. The accumulation of two isoforms of LhcsR protein LhcsR1 and LhcsR3 is different. Recently, it has been found that the expression of LhcsR1 protein is constitutive, while the accumulation of LhcsR3 is increased under EL conditions and depends on the activation of the CAS [55,56] calcium sensor. Upon protonation, *C. reinhardtii* LhcsR subunits switch to a quenching conformation. The dynamics of LhcsR proteins transition between unquenched and quenched conformations has been studied in the moss protein LhcsR1 [57,58,59], showing a 50-fold decrease in lifetime from 3.7 ns lifetime to 80 ps. The physicochemical mechanisms involved were identified to be dual: (i) the transient formation of carotenoid radical cation, thermally recombining to ground state [60,61,62], and (ii) the energy transfer from a Chl *a* to lutein S1 state, which thermally relaxes to the ground state within approx. 10 ps [61]. Thus, the two types of quenching mechanism reported for plants, as localized, respectively, in two different types of LHC subunits [61,63,64], appear to be both active within the single LhcsR subunit [57]. Under EL conditions, lumen acidification triggers the so-called xanthophyll cycle, which involves the xanthophylls violaxanthin (Vio) and zeaxanthin (Zea), and consists of a light-dependent, rapid and reversible de-epoxidation of Vio to Zea. The reaction is catalysed by VDE (violaxanthin de-epoxidase). This enzyme is luminal in plants where it is activated by acidification, while it is stromatic in *Chlamydomonas* [65]; the xanthophyll cycle of intact *Chlamydomonas* cells is inhibited by the uncoupler nigericin, indicating that the activation of this stromal enzyme also requires the build-up of a pH gradient in EL. The amplitude of qE in plants correlates with the level of Zea though its binding to specific LHC targets, in *C. reinhardtii* NPQ amplitude is Zea-independent [66]. The qT component of NPQ is dependent on ST, i.e., the mechanism of LHCII relocation between PSs, which compensates for PSI/PSII excitation imbalance and optimizes photosynthetic electron transport in response to the light conditions. PSII over-excitation reduces PQ to PQH_2_, and activates a thylakoid protein kinase (STT7 in green algae and STN7 in higher plants) which, in turn, phosphorylates LHCII, and leads to its reversible association with the PSI-LHCI complex [67]. In *C. reinhardtii*, most of LhcbM proteins get phosphorylated upon ST, including the monomeric antennae CP29 and CP26, which are recruited as a supplementary antenna for PSI. While this mechanism is widespread in green photosynthetic organisms, in plants the amplitude of ST is lower than in *C. reinhardtii*, possibly indicating differences in the regulation of photosynthetic electron transport. The term “qI” refers to all quenching processes relaxing slowly (>10 min), and comprises multiple processes contributing to the down-regulation, inactivation and damaging of PSII. One of the components of the slowly-relaxing NPQ correlates with the synthesis of Zea, was shown as ΔpH-independent, and is possibly related to the binding of Zea to specific antenna proteins [68]. A second component of NPQ is related to photoinhibition and is enhanced upon prolonged over-excitation. It consists into a light-induced reduction of the quantum yield of PSII, due to the photodamage of the RC protein D1. Thus, quenching relaxation reflects the kinetic of RC repair cycle [20].

**Figure 4:**
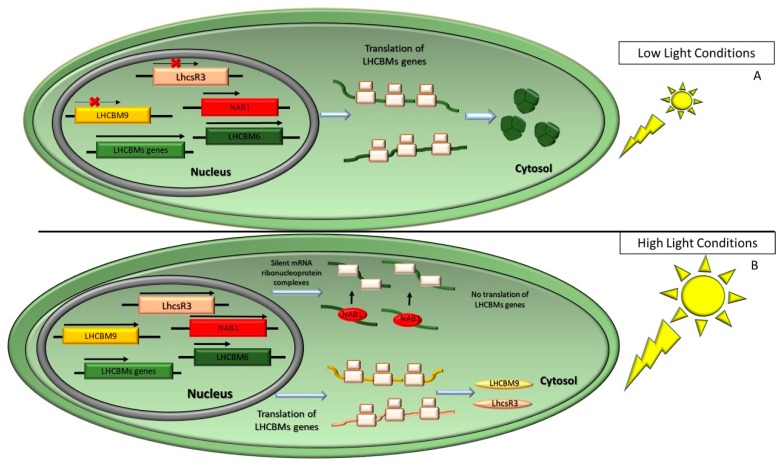
Scheme of long-term control mechanisms regulating light harvesting antenna size, as described in the model alga *C. reinhardtii*. (**A**) In low light conditions, carriers of the photosynthetic electron transport chain are oxidized, and all nuclear genes encoding LhcbMs isoforms associated to the PSII are expressed, except for the isoform 9. LhcbM-encoding mRNAs are translated in the cytosol, then targeted to the chloroplast and inserted in the thylakoid membranes. Under low light conditions, the translational repressor NAB1 is in a less active state. (**B**) In excess light conditions, ATP and NADPH produced by the light reactions exceed their consumption rate by the CBBc, and the overexcitation of PSII results in the release of reactive oxygen species (ROS). To alleviate excitation pressure, a remodelling of the antenna system is induced by slowing down the transcription of LhcbM genes. Once the translation of NAB1 is promoted, this subunit interacts with LhcbM-encoding mRNAs to form silent mRNA-ribonucleoprotein complexes. In contrast to all other isoforms, the expression of LhcbM9 and LhcsR3 proteins are induced.

The NPQ mechanism is highly relevant for the maintenance of the photosynthetic efficiency which contributes to acclimation to the different light environments. The relative contribution of each of the NPQ components changes between organisms and irradiances: qE activates based on sudden increases in light intensity, while ST responds to changes in the light spectrum under low light conditions. Thus, LhcsR protein function is synergically with other photoprotective mechanisms, such as CET and ST in shaping the fast response to environmental conditions. Long-term stresses occur on timescales of days and weeks. Photoacclimation mechanisms to such changes involve a rearrangement at the level of chloroplast protein and lipid composition yielding into an adjustment of the stoichiometry of photosynthetic complexes through the modulation of gene expression and synthesis/degradation of individual chloroplast components. A major component of response to excess light consists into down-modulating the size of the PSII antenna [69], and enhancing the stoichiometry of the Cyt *b*_6_*f* complex, ATPase and RuBisCO with respect to PSII RC. In green algae, EL down-regulates LHCII and LHCI genes transcription, while when under limiting irradiance, the opposite response was shown [70,71]. In *C. reinhardtii* the increase in the Chl *a*/*b* ratio is consistent with decreases in the amount of both LHCI and LHCII in EL [70,72,73]; EL stress also induces an accumulation of proteins involved in the NPQ response, such as LhcsR3 in *C. reinhardtii* [72,74]. Moreover, the LhcbM isoforms are expressed differentially depending on growth conditions, which suggests a specific role of different LHC complexes in PSII organization and chloroplast photoprotection [75,76,77]. LhcbM9 is only expressed in stressing conditions and binds to PSII–LHCII complexes, where it protects PSII by inducing an energy-dissipative state with reduced ^1^O_2_ formation [77]. In *C. reinhardtii*, the transcriptional regulation of *LhcbM* genes is mediated by nucleic acid binding 1 protein (NAB1), a cytosolic protein that prevents the translation of LhcbM by sequestering the corresponding mRNAs into translationally silent ribonucleoprotein complexes [78] (Figure 4). The relative abundance of NAB1 is regulated by nutrient abundance: Under CO_2_ starvation, which hampers the activity of the CBBc, up-regulation of NAB1 promotes an antenna size reduction, thus alleviating the excitation pressure on PSs.

## 3. Improving Photosynthetic Yield

### 3.1. Light Harvesting Antenna as Target to Reduce Optical Density in Mass Culture

**Figure 5:**
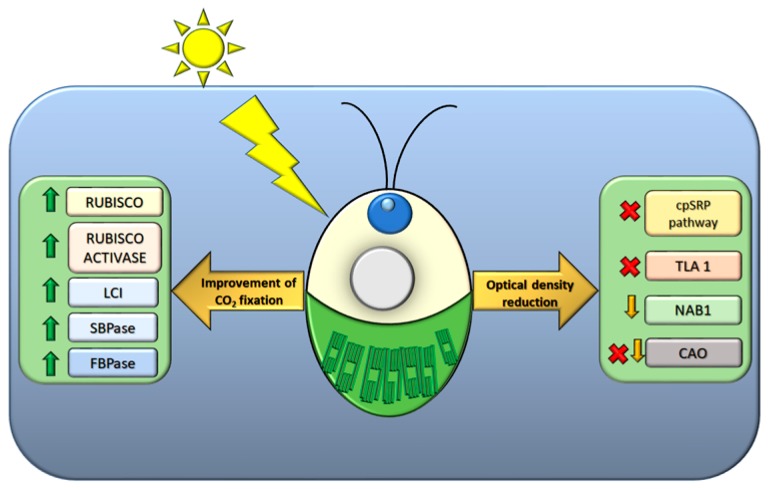
Genes successfully targeted in *C. reinhardtii* or other species to improve photosynthetic productivity. Improvement of CO_2_ fixation targets: RuBisCO, RuBisCO activase, LCI (Low-CO_2_ Inducible protein), SBPase (sedoheptulose1,7-biphosphatase), FBPase (fructose-bisphosphate aldolase). Optical density reduction: cpSRP pathway (chloroplast signal recognition particle), TLA1 (Truncated Light-Harvesting Antenna 1), LhcbM, NAB1 (nucleic acid binding 1 protein) and CAO (Chlorophyllide *a* Oxygenase). Green arrows indicate the over-expressed genes, yellow arrows the down-regulated genes and red crosses indicate the knocked-down genes.

When grown under mass culture, a condition typical of industrial PBRs, microalgae undergoes a progressive drop in productivity as the cell density gradually increases. This can be mainly ascribed to an inhomogeneous light distribution within the culture, due to its high optical density: In this condition, the surface layers of the culture easily reach the saturation of photosynthesis (and possibly photoinhibition), while the inner layers are light-limited. Such steep gradient in light penetration results in a low productivity of the system. Optimization of the light transmittance within the culture volume was proposed as a strategy to alleviate these constraints. A bioengineering approach to decrease the Chl content per cell, thus minimizing the light absorption and enabling a larger fraction of cell suspension to contribute to overall productivity, was first developed in the model alga *C. reinhardtii*. Truncated light-harvesting antenna (*tla*) mutants were obtained by random DNA insertional mutagenesis and selection by Chl fluorescence imaging. Mutant *tla1* showed a significant reduction of Chl content per cell and a lower functional antenna size of both PSI (−50%) and PSII (−65%) vs. wild type (WT) [79]. In batch culture, *tla1* cells yielded a higher P_max_ at saturating irradiances and higher light-to-biomass conversion efficiency with respect to the WT strain [79]. Gene *TLA1* was found to participate in the mechanism of Chl antenna size regulation, and indeed its over-expression resulted in a larger antenna size for both photosystems and lower Chl *a/b* ratio with respect to WT, while its down-regulation by RNAi resulted in the opposite phenotype [80]. Strain *tla2* was mutated in the gene encoding the chloroplast-localized signal recognition particle (CpSRP) receptor CpFTSY, whose deletion was responsible for a pale-green phenotype and a lower Chl *a*/*b* ratio than WT [81]. Components of the CpSRP complex, involved in the proper folding of LHCs and targeting of these proteins to the thylakoids, are therefore promising molecular targets to achieve a substantial reduction in Chl antenna size without impairing the photosynthetic electron transport (Figure 5) [82]. Moreover, CRISPR-Cas9 technology was recently shown as a reliable approach by which to produce *tla* mutants [83,84]. Pale-green mutants were obtained in species other than *C. reinhardtii* (Figure 5): *N. gaditana* and *C. sorokiniana* mutant strains with truncated antenna were isolated by random mutagenesis and phenotypic selection; once characterized, they showed higher photosynthetic efficiency than WT and improved photoresistance under EL conditions, in both lab-scale and industrial-scale PBRs [12,85]. An additional molecular target expected to affect antenna size was *CAO* (encoding for Chlorophyllide *a* oxygenase) (Figure 5), encoding for the enzyme responsible for Chl *a* → Chl *b* conversion [86]. In *Chlamydomonas*, both insertional knock-out and point mutations on *CAO* impaired the biogenesis of antenna systems, which were affected in different ways depending on the light conditions [87]. Moreover, CAO expression was modulated by RNAi, which resulted in knock-down mutants showing a lower Chl *b* content. Therefore, by tuning the Chl *b* relative abundance, corresponding regulation of antennae size can be obtained, and a reduced optical cross-section improves the growth and photosynthetic rate under high light conditions, without impairing other regulatory mechanisms such as ST and NPQ [88].

Additional perspectives towards enhancing the light use efficiency in algae are likely to be developed in the future based on the emerging functional diversity of individual Lhcm proteins which have been reported to be involved in state1–state2 transitions, NPQ and/or in sustained photoprotection [75,77] thus opening the perspective of enhancing such functions selectively in industrial strains. Nevertheless, it is not yet clear how engineering antennas can be combined with the well-established enhanced growth efficiency of truncated antenna strains [12,89].

### 3.2. Bioengineering Response to Light Fluctuations and Improving Resistance to Photo-Inhibition

The capacity to counteract EL stress and avoid photoinhibition clearly provide a carbon-gain advantage and therefore represent an important component of productivity. In particular, responses to fluctuating light conditions are clearly beneficial for photosynthesis since they enhance the ‘tracking’ of light, thus maintaining high rates of C assimilation, as shown in field crops [90]. In microalgae, the WT strains show impaired growth when excess irradiance induces photoinhibition, since the repair of photodamage requires metabolic energy. Engineering of the response to EL succeeded in mitigating this loss in efficiency: Very high light resistant (VHL-R) *Chlamydomonas* strains were selected for their ability to grow at irradiances lethal to the control genotype, and found they were affected in the pathways which regulate photoprotective responses, including PSII repair and ROS detoxification [91]. The *Chlamydomonas* WT strain was UV-mutagenized and selected on a lethal concentration of Red Bengal, a photoreactive chemical releasing ^1^O_2_; characterization of tolerant strains identified SOR1 as a factor enhancing resistance to photoinhibition [92]. Analogously, UV-mutagenesis and selection under high irradiance (2000 μmol photons m^−2^ s^−1^) identified the Light Responsive Signal 1 (LSR1) gene, which conferred improved resistance against exogenous ROS [93]. Recently, Dall’Osto and colleagues [89] applied two steps of mutagenesis and phenotypic selection to *Chlorella vulgaris*. First, they selected a strain characterized by a 50% reduction of Chl content per cell and a 30% increased photon-to-biomass conversion efficiency with respect to WT. After a second mutagenesis cycle followed by a selection on Rose Bengal, they selected pale-green genotypes exhibiting higher resistance to singlet oxygen (strains SOR) that showed a further enhancement in biomass productivity with respect to both parental and WT strains [89].

Alternatively to genetic engineering and mutation/phenotypic selection, an alternative approach consists into sampling and evaluating algal biodiversity, particularly in extreme environments which might provide interesting performance when such strains are grown in optimal conditions. An example of this is the case of *Chlorella ohadii*, a chlorella strain from the Sinai desert, which was reported to exhibit high productivity and the robustness of growth [94,95].

### 3.3. RuBisCO as Target to Improve Carbon Assimilation Efficiency

The rate-limiting step of the CBBc is the fixation of inorganic carbon catalysed by RuBisCO, as the complex has low turnover rate and low substrate specificity. Moreover, it shows affinity for O_2_ which leads to futile reactions. The consequences of the wasteful oxygenation reaction are partially alleviated by the photorespiration process which, nevertheless, yields into a partial loss of the CO2, and thus decreases light-to-biomass conversion efficiency [96]. Therefore, the engineering of microalgal strains with enhanced RuBisCO catalytic activity would be crucial for improving the efficiency of solar energy conversion. Some species of red algae express isoforms with high specific activity [43]. Thus, combining positive mutations from different isoforms has been suggested as a way to obtain RuBisCO with the improved V_max_ of carboxylation catalysis [97]. A major constraint to this approach is the high intolerance of the catalytic region to mutations, that made sparsely successful direct evolution strategies [96]; nevertheless, some enzymes variants with higher activity have been identified, and their heterologous expression represents a promising approach [98]. Other RuBisCO-improved variants were obtained by site directed mutagenesis, targeting either the rbcL gene (RuBisCO large subunit) or the subunit that interacts with Rubisco activase [99,100]. However, their over-expression in *Chlamydomonas* failed to enhance the C fixation efficiency [101]. On the contrary, the over-expression of endogenous RuBisCO activase in *Nannochloropsis oceanica* increased biomass and lipid productivity up to 40% (Figure 5) [102]. Consistently, over-expression of RuBisCO in the cyanobacterium *Synechocystis* enhanced photosynthetic efficiency and fatty acid productivity (Figure 5) [103,104]. In *Chlamydomonas*, a number of strategies were tested to improve carbon assimilation. RuBisCO isoforms with the higher V_max_ of carboxylation catalysis were obtained by the PCR-based gene shuffling of the rbcL gene consisting into a restriction of encoding DNA following low fidelity replication and re-ligating into random assembled sequences with enhanced biochemical variability [105]. The site-directed mutagenesis of rbcL resulted in a low-activity RuBisCO variant, which instead triggered a ten-fold higher H_2_ production in *Chlamydomonas*, possibly by increasing the pool of reducing equivalents available to the hydrogenase [106]. An alternative approach would alter the engineering of cyanobacterial CO_2_-concentrating mechanisms, as a possible route to enhance the RuBisCO operating efficiency. Before the approach delivers potential benefits, characterization of algal HCO_3_^−^ transporters and carbonic anhydrases, and identification of factors regulating RuBisCO aggregation into the pyrenoids, is required. Recent advances in dissecting the details of pyrenoid biogenesis in *Chlamydomonas* [107] might guide future redesign of the mechanism, to augment the overall C fixation rate.

Besides RuBisCO, other CBBc enzymes and accessory proteins have been targeted, e.g., sedoheptulose 1,7-bisphosphatase from *C. reinhardtii* has been successfully over-expressed in *D. bardawil*, resulting in a significant enhancement of photosynthetic efficiency (Figure 5) [108]. Over-expression of the fructose 1,6-bisphosphatase in *Synechocystis* enhanced the growth rate with respect to the control genotype under EL conditions (Figure 5) [109]. A strong raise in the photosynthetic productivity of *Synechocystis* was obtained by over-expressing RuBisCO, sedoheptulose1,7-biphosphatase, fructose-bisphosphate aldolase and trans-ketolase [110].

Finally, by the over-expression of Low-CO_2_ Inducible (LCI) proteins in *C. reinhardtii* maintained at high CO_2_ concentration, namely under conditions which repress LCI synthesis, an increase of biomass productivity up to 80% with respect to the control genotype was reported (Figure 5) [111].

### 3.4. Engineering of the Lipid Biosynthesis for Renewable Energies Production

The triose phosphate produced by photosynthesis supports the main metabolic pathways of the algal cell, therefore the enhancement of photosynthetic yield potentially results in the enhancement of lipids, proteins and other high value compounds synthesis. Genetic manipulation approaches can generate strains with desirable commercial traits, by either expressing new biosynthetic pathways or enhancing the yield of a product of interest already present in a given strain.

The major research targets is the engineering of strains for a significant increase of total lipid accumulation, and/or the optimization of fatty acid chain-length profile, which can be carried out by targeting single or multiple genes involved in the lipid biosynthesis or by down-regulating competing pathways [112,113]. Saturated and mono-unsaturated C_14_–C_20_ fatty acids from microalgae are exploited for renewable liquid biofuel production, while the engineering biosynthesis of long chain-polyunsaturated fatty acids (LC-PUFAs), important components of the human diet, might become a viable option in the market of high-value food additives [114]. Metabolic engineering reports for redirecting carbon fluxes toward fatty acid production in microalgae, included the up-regulation of key biosynthetic enzymes: (i) acetyl-CoA carboxylase, catalysing the first step for fatty acid biosynthesis, was successfully over-expressed in the chloroplast of *P. tricornutum* [115]; (ii) malonyl-CoA ACP transacylase enzyme, which catalyses the formation of malonyl-ACP, was over-expressed in both *N. oceanica* and *Schizochytrium spp.*, resulting in 31% and 39.6% total lipid increase with respect to the control genotype, respectively [116,117]; (iii) acyl-ACP thioesterases (TE), involved in the last step of fatty acids biosynthesis, was heterologously expressed in different strains of microalgae [118,119]. In *Dunaliella tertiolecta*, a combinational expression platform involving plant lauric acid-TE (C12TE) and medium-chain fatty acid-specific ketoacyl-ACP synthase was recently engineered, and resulted in a significant increase in lauric acid (C12:0) and myristic acid (C14:0) accumulation [120].

In addition to the strategies for increasing lipid production, fatty acid desaturation could be regulated in order to modify the PUFAs profile, by engineering either the desaturase/elongase pathway or the polyketide synthase pathway [121]. Both endogenous and exogenous desaturases and elongases have been successfully over-expressed in different oleaginous microalgae such as *N. oceanica* and *P. tricornutum* [122,123,124]. In *Chlamydomonas*, over-expression of the endogenous CrΔ4FAD, a monogalactosyl-diacylglycerol Δ4-desaturase, resulted in increased production of the specific product hexadeca-4,7,10,13-tetraenoicacid (16:4) [125].

Microalgal lipid composition differs among species, moreover it is further affected by the cultivation conditions. The main storage lipids in oleaginous microalgae are triacylglycerols (TAG), which are more desirable for commercial-scale biodiesel processing than polar lipids or free fatty acids [126,127]. The first step of TAG biosynthesis is catalysed by acyl-CoA: glycerol-3-phosphate acyl-transferase, whose over-expression in *P. tricornutum* was shown to promote the formation of oil bodies, and led to a significant increase in lipid content [128,129]. A significant increase in lipid productivity was also obtained by up-regulating endogenous lysophosphatidic acid acyltransferase (LPAAT) both in *C. reinhardtii* and in *P. tricornutum*, and by over-expressing the *Brassica napus* LPAAT isoform in *Chlamydomonas* [130,131,132]. An additional rate-limiting steps in the TAG biosynthesis of microalgae is catalysed by chloroplastic (DGAT1) and ER (DGAT2) isoforms of diacylglycerol acyltransferase (DGAT) [133]. Over-expression of DGAT1/DGAT2 in *P. tricornutum* and *Nannochloropsis spp.* resulted in a significant increase of total lipid accumulation [128,134,135,136].

Other enzymes which have been targeted by genetic engineering approaches, and which have succeeded in improving TAG content, included that which was involved in acetyl-CoA synthesis: (i) the *E. coli* isoform of ACS (acetyl-CoA synthetase) was introduced in *Schizochyrium* [137]; (ii) PDC (pyruvate dehydrogenase complex) abundance was increased by down-regulating pyruvate dehydrogenase kinase (PDK) in *P. tricornutum* [138]; malic enzyme (ME) and glucose-6-phosphate dehydrogenase (G6PDH) were over-expressed in *P. tricornutum* [139,140].

In addition to the modification of fatty acid biosynthesis, competitive pathways e.g., starch synthesis have also been manipulated for generating wider metabolic changes [141]. The conversion of phosphoenolpyruvate (PEP) to oxaloacetate by PEPC (PEP carboxylase) preferentially diverts the carbon flow towards protein synthesis, and indeed PEPC down-regulation resulted in the over-expression of pyruvate kinase, which increased the levels of pyruvate and acetyl-CoA [142]. On the other hand, knock-down of a PEPC isoform by either artificial microRNA-mediated technology and CRISPRi in *Chlamydomonas*, and by RNAi in *P. tricornutum*, resulted in higher TAG accumulation [112,143,144].

Another strategy to improve TAG production is the inhibition of specific lipases. In *Nannochloropsis oceanica* the TAG lipases mutants *tgl1* and *tgl1;2* increased the TAG content by two-fold in early log phase cells [145].

Transcription factors represent a suitable target to be manipulated as an alternative to structural genes, since they proved to be more effective for inducing global metabolic changes. Several transcription factors controlling lipid biosynthetic pathways have been identified, and their over-expression resulted into more productive strains. The up-regulation of a Dof-type transcription factor in *Chlamydomonas* doubled the cellular content of TAG [146,147]. Over-expression of bZIP and bHLH transcription factors in both *N. salina* and *N. oceanica* improved biomass and lipid productivity [148,149,150,151]. In *N. gaditana*, 20 putative regulators of lipid production, down-regulated by nitrogen deprivation, were identified by RNA-seq; a strategy of selective knock-out by the Crispr-Cas9 system allowed us to identify a homolog of the fungal Zn(II)_2_Cys_6_-encoding gene, which triggered lipid biosynthesis in *Nannochloropsis* and resulted in a 200% increase of C partition to lipids, without significantly affecting the growth rate [152]. More recently, a Myb-like transcription factor Phosphorus Starvation Response (PtPSR) was identified in *P. tricornutum*; its modulation might represent a good strategy to enhance cell growth and TAG production in limited-phosphorous conditions [153]. A MYB DNA binding protein involved in cell cycle regulation was instead targeted in *Chlamydomonas*, showing that the mutants devoid of CrCDC5 accumulate more oil and starch with respect to WT [154].

Integrated, multiomic analysis has been recently performed on different high-lipid productive microalgal strains, in order to pinpoint a range of candidate molecular targets, aimed to enhance oil productivity in different growth conditions [155,156].

### 3.5. Endogenous Up-Regulation and Heterologous Expression of Isoprenoid Biosynthetic Pathways in Microalgae

The photoautotrophic nature of microalgae requires the generation of isoprenoids, natural hydrocarbons associated with the photosynthetic apparatus (e.g., carotenoids, plastoquinon, phytol chains) and participating in photon capture and electron transport events. Thus, microalgae own specific isoprenoid biosynthetic pathways that could be manipulated by genetic engineering to increase the natural capacities for the generation of isoprenoids, or to promote heterologous isoprenoid production. These class of molecules, due to their intensive color, fragrance and antioxidant properties, are in high demand for various applications in animal feed, medicines and nutraceuticals, pest control agents, cosmetics and pigments [157].

Regarding the endogenous isoprenoids, carotenoids represent commercially successful products from microalgae: β-carotene from *D. salina* and astaxanthin from *H. pluvialis* are high-price products mainly used in aquaculture, and as food colouring agents and nutraceuticals [158]. As for zeaxanthin, hyper-accumulating *D. salina* strains were identified [159], while a ΔZEP *Chlamydomonas* strain with a far higher zeaxanthin content than WT (56-fold) was shown to have a commercial potential in the production of eggs fortified with carotenoids [83,160]. In *H. pluvialis*, the endogenous phytoene desaturase was modified by site-directed mutagenesis, enhancing both the resistance to the herbicide norflurazon and the astaxanthin productivity [161]. Significant enhancements in carotenoid productivity were obtained by either over-expressing the β-carotene ketolase or expressing the endogenous phytoene desaturase in the chloroplast under the control of the psbA promoter [162,163].

In *Chlamydomonas*, the over-expression of *C. zofingiensis* ZEP resulted in an astaxanthin production, reaching values of 0.5 mg g^−1^ DW [164]. To increase the production of fucoxanthin, employed as an antioxidant agent, the 1-deoxy-d-xylulose 5-phosphate synthase and the phytoene synthase were over-expressed in *Phaeodacthylum* [165].

Two pathways lead to synthesis isoprenoid products: The 2-c-methyl-D-erythritol 4-phosphate/1 deoxy-D-xylulose 5-phosphate (MEP/DOXP) pathway and the mevalonate pathway (MVA). These are localized in different cell compartments but generate the same 5-C precursors, isopentenyl diphosphate (IPP) and dimethylallyl diphosphate (DMAPP). The MVA pathway was lost during evolution in the Chlorophyta and Eustigmatophyceae [166], while it was conserved in other microalgae, e.g., *P. Tricornutum* maintained the cytosolic MVA pathway, thus representing a choice species for isoprenoid engineering and heterologous terpenoid production [157,166].

In *Phaeodacthylum*, lupeol synthase isoforms from *A. thaliana* and *L. japonicus* were expressed to produce lupeol, an anti-inflammatory triterpene [157]. Moreover, co-expression in the diatom of CYP716A12 from *M. truncatula* and its corresponding reductase MtCPR, was aimed to further functionalize lupeol in betulin for the production of the antiretroviral, antimalarial agent betulinic acid [157]. In *Chlamydomonas*, the fragrance patchoulol was produced by expressing the patchoulol synthase from *Pogostemon cablin* [167], while the synthesis of bisabolene, a sesquiterpene compound identified as a promising jet fuel candidate, resulted by both over-expressing the bisabolene synthase from *Abies grandis* and down-regulating both the geranylgeranyl diphosphate (GGPP) synthase and the squalene synthase [168].

Finally, several non-native diterpenoids were successfully produced in *C. reinhardtii*, by the heterologous expression of diterpene synthases targeted to the algal chloroplast, to convert native GGPP into the diterpenoid products casbene, taxadiene and 13R(+) manoyl oxide [169].

Notably, besides diatoms and green microalgae, Cyanobacteria represent the most targeted photosynthetic organisms for isoprenoid production, especially terpenoids [170]. Cyanobacteria are, indeed, promising platforms for biofuel production [171], and the knowledge on isoprenoid biosynthetic pathways regulation is instrumental in over-expressing key enzymes toward a higher productivity. To enhance isoprene synthesis, different plant isoprene synthase (IS) genes were expressed in *Synechococcus elongatus*, in particular the IS from *Eucalyptus globulus* was responsible for the most significant productivity enhancement among the isoforms tested [172]. Moreover, limonene is a terpenoid evaluated as a promising alternative fuel. The limonene synthase from *Mentha spicata* was successfully expressed in different species such as *Synechococcus elongatus* PCC 7942, *Synechocystis* sp. PCC 6803 and *Synechococcus sp.* PCC 7002 [173,174,175].

## 4. Concluding Remarks

Nowadays the technical advance in genetic engineering made it possible to engineer algal strains for enhancing both biomass productivity and the yield of high value products from microalgae. The major efforts are focused on obtaining strains with higher photosynthetic efficiency in order to decrease the unitary cost of algal biomass. The best results have been obtained by enhancing the homogeneity of light distribution within photobioreactors [82] and by decreasing their susceptibility to photodamage [89]. Besides the obvious strategy of targeting antenna systems, photoprotection was also enhanced by increasing photochemical quenching by boosting CO_2_ fixation through over-expressing RuBisCO activity, and other rate-liming enzymes in the CBB cycle [109].

Besides productivity, microalgae exploitation targets high value compounds. First, oleaginous strains, especially diatoms, were engineered to optimize the fatty acid chain-length profile for biodiesel production [141]. Secondly, isoprenoid biosynthesis pathway has been engineered to redirect carbon flux towards specific and high values products for chemical industry and/or as biofuels [176].

While the overall strategies have been well defined, limitations derive mainly from the low number of species that can be engineered to some extent besides the model species *Chlamydomonas reinhardtii* and the efficiency of the expression of the transgenes in different species [177].
